# Tibial Acceleration-Based Prediction of Maximal Vertical Loading Rate During Overground Running: A Machine Learning Approach

**DOI:** 10.3389/fbioe.2020.00033

**Published:** 2020-02-04

**Authors:** Rud Derie, Pieter Robberechts, Pieter Van den Berghe, Joeri Gerlo, Dirk De Clercq, Veerle Segers, Jesse Davis

**Affiliations:** ^1^Department of Movement and Sports Sciences, Ghent University, Ghent, Belgium; ^2^Department of Computer Science, KU Leuven, Leuven, Belgium

**Keywords:** running biomechanics, impact loading, tibial shock, machine learning, wearable sensor, gait analysis

## Abstract

Ground reaction forces are often used by sport scientists and clinicians to analyze the mechanical risk-factors of running related injuries or athletic performance during a running analysis. An interesting ground reaction force-derived variable to track is the maximal vertical instantaneous loading rate (VILR). This impact characteristic is traditionally derived from a fixed force platform, but wearable inertial sensors nowadays might approximate its magnitude while running outside the lab. The time-discrete axial peak tibial acceleration (APTA) has been proposed as a good surrogate that can be measured using wearable accelerometers in the field. This paper explores the hypothesis that applying machine learning to time continuous data (generated from bilateral tri-axial shin mounted accelerometers) would result in a more accurate estimation of the VILR. Therefore, the purpose of this study was to evaluate the performance of accelerometer-based predictions of the VILR with various machine learning models trained on data of 93 rearfoot runners. A subject-dependent gradient boosted regression trees (XGB) model provided the most accurate estimates (mean absolute error: 5.39 ± 2.04 BW⋅s^–1^, mean absolute percentage error: 6.08%). A similar subject-independent model had a mean absolute error of 12.41 ± 7.90 BW⋅s^–1^ (mean absolute percentage error: 11.09%). All of our models had a stronger correlation with the VILR than the APTA (*p* < 0.01), indicating that multiple 3D acceleration features in a learning setting showed the highest accuracy in predicting the lab-based impact loading compared to APTA.

## Introduction

Ground reaction forces are relevant parameters for running analysis (Pohl et al., 2009; Crowell and Davis, 2011; Van Der Worp et al., 2016; Clark et al., 2017). They partially describe the center of mass’ state of motion during running and are often used by sport scientists and clinicians to analyze the mechanical risk-factors of running related injuries (Bredeweg et al., 2013; Napier et al., 2018) and/or athletic performance (Preece et al., 2019).

A commonly used ground reaction force-derived variable is the maximal vertical instantaneous loading rate (VILR), which is calculated as the maximal slope of the rising vertical ground reaction force – time curve (Ueda et al., 2016). VILR has been used to characterize the impact (i.e., high rate of force development due to the rapid deceleration of all body segments during the foot-ground collision) during running (Gerritsen et al., 1995). This measure could discriminate groups of rearfoot runners with a history of stress fractures (Van Der Worp et al., 2016) and plantar fasciitis (Pohl et al., 2009). Consequently, VILR has been considered clinically relevant and has been a main outcome variable in gait retraining studies targeting runners with high VILR (Crowell and Davis, 2011; Clansey et al., 2014; Willy et al., 2016).

Ground reaction forces are traditionally measured using fixed force platforms or instrumented treadmills (Ueda et al., 2016). Unfortunately, measurements with force platforms are laboratory-based and require both expensive equipment and extensive post-processing. These factors limit the potential of monitoring in-field running biomechanics, whereas wearable inertial measurement units can accommodate this by predicting running gait parameters outside the laboratory (Falbriard et al., 2018; Wouda et al., 2018). In this respect, an ambulatory low-cost accelerometer was proposed as a potential surrogate candidate to estimate VILR when force platforms are not available (Ngoh et al., 2018). Previous research has identified a moderate to good correlation (range of *r*_mean_ = 0.64–0.84) between the axial peak tibial acceleration (APTA) captured by a skin-mounted accelerometer at the tibia and VILR (Laughton et al., 2003; Pohl et al., 2009; Greenhalgh et al., 2012; Zhang et al., 2016; Van den Berghe et al., 2019). Therefore, using APTA as a surrogate measure for VILR seems justifiable (Sheerin et al., 2019).

However, the APTA is based on a single, basic feature (i.e., the peak value) of the time-continuous 1D tibial acceleration signal. Consequently, a large amount of data is neglected, which may lead to missing important information. A combination of multiple features of the 3D tibial acceleration signals, possibly including complex and higher-order ones, may result in a more accurate predictor of VILR than only considering APTA. Hence, a performant computational model that extracts relevant features and effectively copes with any non-linear relationships (between the features of the tibial acceleration signals and the target VILR) is desired. In that way, machine learning techniques could help to analyze continuous time-series data without pre-selecting discrete variables. Holzreiter and Köhle (1993) introduced the use of neural networks to assess gait patterns in locomotion biomechanics. Recently more advanced machine learning techniques have been used to detect pathologic gait-patterns (Williams et al., 2015; Zeng et al., 2016), fatigue (Janssen et al., 2011; Op De Beéck et al., 2018) as well as classifying gender, performance-level (Clermont et al., 2018) and age-related running patterns (Fukuchi et al., 2011).

To gain a better understanding of the relationship between the external load and potential injury risk in overground running, a more accurate estimation of the athlete’s impact loading is an essential methodological prerequisite. The screening of runners on impact intensity could be more accurate by estimating VILR by means of a machine-learned model instead of relying on the APTA only. Consequently, this study proposes and evaluates the performance (e.g., predictive accuracy, calculation time, diagnostic ability) of an inertial sensor-based method to estimate the runner’s VILR based on bilateral 3D shin-mounted accelerometer data using a machine learning approach. It was hypothesized that the incorporation of these extracted features into a set of machine-learned models would result in stronger predictive and diagnostic capacities than considering APTA only.

## Materials and Methods

### Ethics Statement and Participants

Ninety three subjects engaged in recreational as well as competitive running (55 men and 38 women) were recruited from the local community. Runners were included if they were free of running-related injuries and ran at least 15 km per week (Table 1). All subjects signed an informed consent prior to the testing. Approval for the study was obtained from the ethical committee of the Ghent University Hospital (2015/0864).

**TABLE 1:** Characteristics of the subjects.

	**Men**		**Women**	
	**Mean**	***SD***	**Mean**	***SD***
Age (Yrs.)	35.9	9.2	34.6	10.8
Body height (m)	1.79	0.07	1.67	0.06
Body mass (kg)	76.5	10.2	60.6	7.3
Training volume (km/week)	36.4	16.9	27.9	11.0

### Protocol and Setup

All runners were equipped with a backpack/tablet system to measure the tibial accelerations (Van den Berghe et al., 2019). Two tri-axial accelerometers (LIS331, Sparfkun, Colorado, United States; 1000 Hz/axis), were as tight as tolerable strapped with sports tape on the antero-medial side of both tibias, 8 cm above the malleolus medialis (Laughton et al., 2003; Clansey et al., 2014). The axis of each accelerometer was orientated in a way that the vertical axis of the accelerometer coincided with the longitudinal axis of the concerned tibia. The skin around the lower leg was pre-stretched with sports tape to improve the rigid coupling between the accelerometers and the tibia (Clansey et al., 2014; Van den Berghe et al., 2019). Data collection took place during two different projects, but with an exact same measurement setup.

**FIGURE 1:**
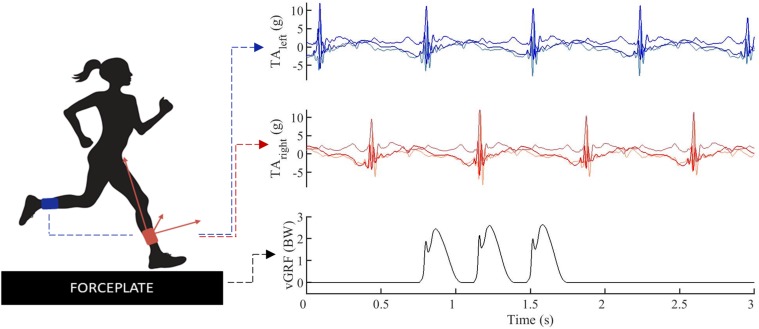
Data example, tri-axial.1pc accelerations (TA) were simultaneously captured for the left (blue) and right (red) lower leg, vertical ground reaction forces (vGRF) were synchronized in time (black).

The first cohort consisted of 13 subjects who were asked to run on a 30 m instrumented running track at multiple running speeds (2.55 ms^–1^, 3.20 ms^–1^, 5.10 ms^–1^, and preferred running speed). All subjects were habitual rearfoot strikers and were provided with the same standardized neutral distance running shoe (Li Ning Magne, ARHF041). The second cohort consisted of 80 runners running at 3.20 m⋅s^–1^. Subjects were not pre-selected on their habitual footstrike pattern and received no verbal instruction about the desired footfall pattern. They wore their regular training shoes. In both cohorts running speed was controlled by timing gates. Recorded trials were discarded and the runners received verbal feedback if their running speed was not within a 0.2 m⋅s^–1^ of the targeted speed. Ground reaction forces were measured at 1000 Hz by two built-in force platforms (2 and 1.2 m, AMTI, Watertown, MA, United States). Accelerometer and force data were synchronized in time (Figure 1) by means of an infrared impulse sent from the motion capture system. The pulse was captured by an infrared sensor attached to the backpack system. For a more detailed description of this synchronization protocol we refer to Van den Berghe et al. (2019).

### Data Processing

#### Example Construction and Data Preprocessing

**FIGURE 2:**
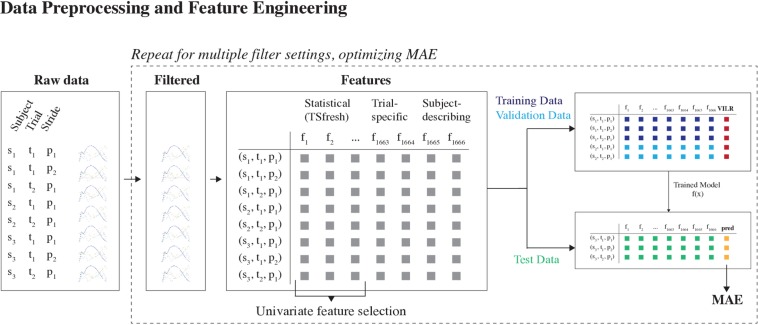
Data preprocessing and feature engineering part of the machine learning pipeline. First, the raw acceleration signals were filtered using a Butterworth bandpass filter. The optimal filter configuration was determined by training multiple models, using different filter configurations. The configuration which enabled the most accurate predictions was used henceforth. Second, feature engineering was used to derive a lower-dimensional representation of the data. The generated features were a combination of automatically generated statistical features and manually crafted domain-specific features. The set of automatically generated features was reduced using a univariate feature selection technique.

Ground reaction force data were filtered using a zero-lag second-order low-pass Butterworth filter with a cutoff frequency of 60 Hz. VILR was calculated as the maximal value of the first derivative of the vertical ground reaction force component following initial contact (vertical ground reaction forces exceeding a 5N threshold) (Ueda et al., 2016). This was subsequently normalized to the subject’s body weight. The acceleration signals were filtered in order to separate the linear acceleration from the gravity component and remove high-frequency noise using the approach of van Hees et al. (2013). The filtering settings were selected using a tuning procedure where 2/3 of the data was used to train a model and 1/3 to evaluate the model. First, to find a sensible range for the parameters, a manual exploration was performed using Chebyshev (type I and type II) and Butterworth filters with settings derived from related research. Subsequently, a grid search of Butterworth filters [(0.2, 1.0; step = 0.2)×(40.0, 70.0; step = 5)] was applied to the acceleration signals and the filter which resulted in the best performance on the evaluation set was selected, which was a second-order band-pass filter with cutoff frequencies of 0.8 and 45 Hz (Figure 2).

We extracted individual strides by splitting the collected signals at the take-off events of the opposite feet. This guarantees that each window contains the part of the acceleration signal that is relevant for determining the VILR. Next, we mirrored the data from the right and left leg, such that each of these strides starts with the right leg making ground contact. This procedure effectively doubled the amount of training data.

Each of the 93 subjects completed on average 16 trials (range: 6 to 67 trials), with each trial containing 2.67 strides on average. In total, 23 trials were removed from the data set due to clear errors in measured ground reaction forces and/or tibial accelerations. This resulted in 4037 examples in total.

#### Feature Construction

A large set of features consisting of three broad categories was considered: (1) auto-generated statistical features of the 3D acceleration waveforms, (2) trial-specific features, and (3) subject-describing features (Figure 2).

##### Auto-generated statistical features

First, from the tri-axial filtered acceleration signals of both feet, we extracted the window between the initial ground contact event and the event where the vertical acceleration component reaches 0 g. Next, we calculated a comprehensive set of time-series features from these windows using the TsFresh Python package (Christ et al., 2018). The extracted features include both basic characteristics of the signals (e.g., mean, maximum, number of peaks, timing of peak values) and more complex features (e.g., continuous wavelet coefficients, coefficients of an autoregressive model, the time reversal symmetry statistic, Fourier coefficients). We refer to the TsFresh paper (Christ et al., 2018) for a full description and list of features.

The FRESH procedure (Christ et al., 2017) was used for feature selection. First, this procedure evaluates the influence of every feature on the target (VILR) using a univariate test (i.e., Kendall rank test for real-valued features and Kolmogorov-Smirnov for binary features) and computes the *p*-value. So, it tests whether the feature and the target are not statistically independent. Subsequently, the Benjamini-Yekutieli procedure was carried out to control for the false discovery rate. This procedure reduced the set of auto-generated features to 1662.

##### Trial-specific features

Running speed, derived from timing gates (Van den Berghe et al., 2019), and ground contact time, derived from tibial accelerations, were included as trial-specific features for each stride. Because the ground contact time cannot be inferred directly from the tibial acceleration signals, we modeled this as a separate prediction problem. Specifically, we solved the related task of predicting the timings of the initial contact and toe off gait events. The ground contact time can then be inferred from the time difference between both events. Due to the interrelations between both gait events (e.g., a toe off event follows 160 to 350 ms after an initial contact event), we framed this as a structured prediction task. In this framework, a function between the acceleration profile and a sequence of initial contact and toe off timings was learned. Specifically, a deep structured recurrent neural network architecture was used. The neural network component of the model used the raw acceleration signals, the jerk (first order derivative of acceleration signals), roll (*arctan*⁡(*a*_*y*_⋅*a*_*z*_)) and pitch ($\arctan\left(-a_{x}\,\sqrt{a_{y}^{2}+a_{z}^{2}}\right)$) of both legs to infer the likelihood of a gait event happening for each sample. Subsequently, the structured component consisted of a constrained peak detection algorithm on the likelihood function that finds the most likely combination of initial contact and toe off timings. Both components were optimized jointly. For a detailed description of this model, see Robberechts et al. (2019).

##### Subject-describing features

Third, the body weight and the shoe type were included. The weight of each subject is a logical feature to consider since the loading rate is expressed as a function of body weight. Furthermore, earlier research has found that footwear properties may affect VILR, even with similar foot-strike patterns (Kulmala et al., 2018). When testing the second cohort (*n* = 80), the subjects reported their shoe brand and type. The shoe’s properties were verified through online databases (running shoes guru, solereview, runner’s world, manufacturer’s website, etc.) and subsequently categorized as being neutral, stabilization or racing flats.

#### Learning Approach

**FIGURE 3:**
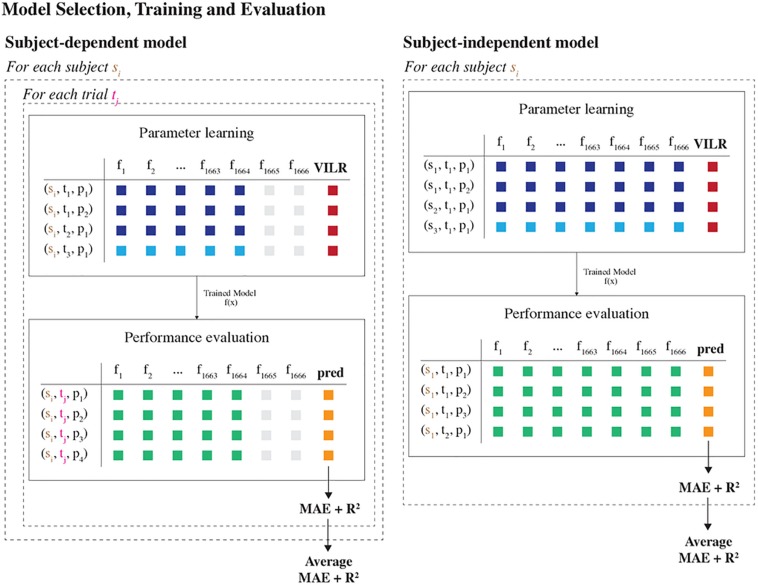
Model selection, training and evaluation part of the machine learning pipeline. Two different learning settings were considered, differing in how the data were split into training and test sets. In the subject-dependent setting, we trained a model on the data of one specific runner, including all trials except one. Subsequently, the model was evaluated on the data of the one held-aside trial. In the subject-independent setting, we trained a model on the data of all runners except for one and evaluated the model on all data of the one held-aside runner. This procedure was repeated for each trial and subject, such that we obtained performance metrics for each fold. Last, the average MAE and R^2^ score per subject were reported.

We considered two different learning settings, each learned on different subsets of the data (Figure 3):

##### Subject-independent model

This setting trained a model using the data from all runners except for one. The model was then evaluated on the trials from the one held-aside runner. That is, at training time the model has no access to any data about the runner for whom predictions will be made. As such, this setup estimates the model’s accuracy when making predictions for new runners for whom there is no available data, which is interesting in practice. Moreover, the model remains valid if a runner adapts his running style.

##### Subject-dependent model

This setting trained a unique personalized model for each subject using only data from that subject. This model would work well if the relationship between the tibial acceleration and the VILR is unique to each subject.

For both settings, we compared the performance of three regression techniques: (1) Linear Regression with Elastic Net regularization (EN), (2) Linear Regression with Least Absolute Shrinkage and Selection Operator regularization (LASSO) and (3) Gradient Boosted Regression Trees (XGB). We used the implementations available in scikit-learn (Pedregosa et al., 2012) for the first two models. For the third regression technique, we used the XGBoost Python package (Chen and Guestrin, 2016).

All models were trained and evaluated in a leave-one-out cross-validation analysis. The subject independent model was iteratively trained on all but one of the subjects to be evaluated on the remaining subject. Similarly, the subject-dependent model is trained on all but one trial of the same subject to be evaluated on the remaining trial. This procedure was repeated for all possible subjects and trials, and the mean accuracy across all folds is reported. As such, this procedure determines the average performance of the models on a group level.

#### Model Evaluation and Statistical Analysis

The model’s accuracy was assessed using both the mean absolute error (MAE) and the coefficient of determination (R^2^ score). The MAE was calculated as the absolute difference between the force platform based VILR and the machine learning predicted VILR. It measures the average magnitude of the errors in the same unit as the VILR and is therefore an easily interpretable measure for the quality of a model. This metric is mainly useful to compare across two models and for domain experts that have insight into the range of VILR values and the magnitude of acceptable errors. The R^2^ score was computed as $R^{2}=1-\frac{\sum_{i}y_{i}-f_{i}}{\sum_{i}y_{i}-\bar{y}}$, where *y*_*i*_ are the force plate based VILR values and *f*_*i*_ are the machine learning predicted values. It has the advantage of being scale-free, thereby indicating how a model performs compared to a constant baseline.

The number of trials completed by each runner varies substantially. In order to avoid that one runner has an excessively large influence on the accuracy of our models, we computed the global MAE and R^2^ score in a two-step procedure. First, the average MAE and R^2^ score were calculated over all strides of that runner. Second, the global metrics were then calculated as the average values of these metrics over all runners that completed at least ten trials. This helps prevent the results from being unduly influenced by a single trial or a single runner.

Additionally, we considered two baseline models: a first model that always predicts a runner’s average VILR for the corresponding landing foot; and a second linear regression model that only includes the APTA as a covariate.

Repeated measures analysis of variance (ANOVA) was used to examine the effect of various learning settings and regression techniques on the estimated VILR. *Post hoc* testing was conducted using a Tuckey HSD test on the relative errors. Additionally, Cohen’s d_*rm*_ effect sizes (Lakens, 2013) were computed for the differences in MAE between each machine learning model and the APTA baseline model. We refer to effect sizes as small (*d*≤ 0.2), medium (0.2 < *d*≤ 0.8) and large (*d* >  0.8) as suggested by Cohen (2013). Statistical analysis was done in Python using the *SciPy* (ANOVA) and *statsmodels* (Tuckey HSD) libraries, with the significance level set at *p* = 0.05.

To assess the diagnostic ability of each model, it was opted to express the model accuracy in the proportion of correct classifications of high impact runners at a common running speed. Because a cut-off for high impact running at the speed of 3.2 m⋅s^–1^ was lacking, those runners with a mean VILR within the highest 33% of our database were selected. The diagnostic ability of the models was assessed by calculating their sensitivity and specificity. Sensitivity is the proportion of runners who are correctly categorized as having a high VILR among those who truly have a high VILR. Similarly, specificity is the proportion of runners who are correctly categorized as not having a high VILR among all runners who truly do not have a high VILR. The Receiver Operating Characteristic curves were constructed to demonstrate the trade-off between both metrics using various cut-off values for the predictions.

## Results

### Predictive Performance of the Machine Learning Models

Table 2 summarizes the predictive performance (MAE and R^2^ scores) of all learned models. In terms of regression techniques, XGB consistently outperformed the other learners (*p* < 0.05; in all but the subject-independent model with subject-describing features setting). Therefore, the results of the XGB learner is reported in the remainder of this section. The differences between the different learning settings were all statistically significant (*p* < 0.05). A subject-independent model without subject-describing features resulted in the least accurate estimations of VILR (MAE: 12.71 ± 7.57 BW⋅s^–1^; R^2^: 0.7397). Including the subject’s weight and shoe type improved the subject-independent model (MAE: 12.41 ± 7.90 BW⋅s^–1^; R^2^: 0.7741). Training a unique model for each subject further improved the predictions by a significant margin (MAE: 5.39 ± 2.04 BW⋅s^–1^; R^2^: 0.9461; *p* < 0.01).

**TABLE 2:** Mean absolute error (MAE) ±SD, coefficient of determination R^2^ scores and effect sizes of MAE’s versus the axial peak tibial acceleration (APTA) baseline for the estimation of the vertical instantaneous loading rate (VILR) by three different regression models.

**Model**	**MAE [BW⋅s^–1^]**	**R^2^**	**d_*rm*_**	**Effect size**
**Subject-independent (without subject-describing features)**				
APTA	21.07 ± 8.13	0.6027	/	
LASSO	13.13 ± 8.79	0.7789	0.3576	Medium
EN	12.91 ± 7.73	0.7811	0.3749	Medium
XGB	12.71 ± 7.57	0.7397	0.4187	Medium
**Subject-independent (with subject-describing features)**				
APTA	18.68 ± 8.44	0.6090	/	
LASSO	12.75 ± 9.01	0.7682	0.3468	Medium
EN	12.48 ± 8.28	0.7713	0.3707	Medium
XGB	12.41 ± 7.90	0.7741	0.4061	Medium
**Subject-dependent**				
APTA	7.39 ± 4.03	0.8500	/	
LASSO	7.50 ± 3.45	0.8657	0.0168	Small
EN	7.36 ± 3.40	0.9124	0.0719	Small
XGB	5.39 ± 2.04	0.9461	0.2900	Medium

*Linear Regression with Elastic Net regularization (EN), Linear Regression with Least Absolute Shrinkage and Selection Operator regularization (LASSO), and Gradient Boosted Regression Trees (XGB) in the subject-independent and subject-dependent learning settings.*

### Predictive Performance of the Single Metric Linear Regression Models

Table 3 shows the predictive performance of linear models that include a single feature in the subject-independent model learning setting. For comparison purposes was the predictive performance of the subject-independent XGB model added as well. Notwithstanding the moderate correlation between the APTA and the VILR, 32 of the extracted features had a higher predictive accuracy than the currently used proxy. Of these 32 features, the mean over the absolute differences between subsequent values of the vertical acceleration signal had the highest correlation with the VILR. A comprehensive overview of all 32 features was made available (Supplementary Table A). The previously discussed regression models that combine multiple of these features still outperform these single-feature models by a large margin.

**TABLE 3:** Mean absolute error (MAE) ±SD and coefficient of determination R^2^ scores for the estimation of the VILR by linear regression models using a single variable in the subject-independent model (SIM) learning setting.

**Statistical model**	**MAE**	**R^2^**
APTA	21.07 ± 8.13	0.60
Standard deviation on linear trend	18.06 ± 7.28	0.67
Mean over the absolute differences between subsequent acceleration values	17.47 ± 7.98	0.71
SIM XGB model	12.41 ± 7.90	0.77

### Diagnostic Ability

**FIGURE 4:**
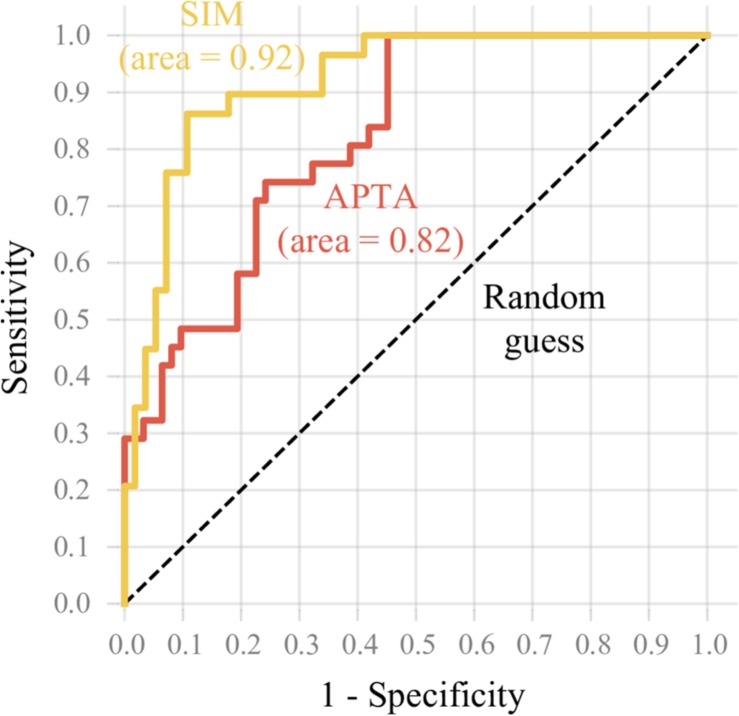
The Receiver Operating Characteristic curve reflects the ability of the subject-independent model XGB (SIM) and APTA models to identify runners with a high VILR. The sensitivity was plotted in function of the false positive rate (1 – specificity). The subject-independent model XGB model had a stronger diagnostic ability than the APTA.

The models’ ability to identify runners with a high VILR is shown in Figure 4. With an area under the curve of 0.92, the subject-independent model XGB had a stronger diagnostic ability than the APTA which has an area under the curve of only 0.82.

**FIGURE 5:**
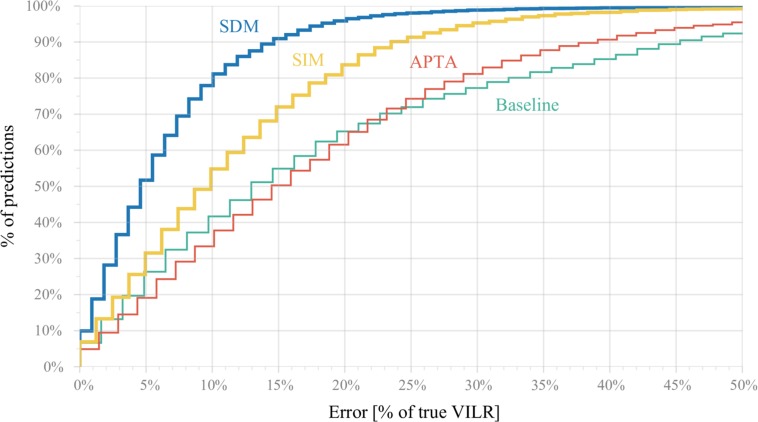
Cumulative percentage (*y*-axis) of predictions for which the relative error is below a threshold (*x*-axis). For example, a relative error of at most 20% on the true VILR can be achieved for 97% of all predictions using the subject-dependent model (SDM), 83% of all strides using the subject-independent model (SIM), 66% using the APTA and 65% by predicting a runner’s average max VILR for the corresponding landing foot (baseline model). The subject-independent model outperformed the baseline and APTA by a significant margin. Similarly, the subject-dependent model outperformed all others, but is less applicable in practice.

Figure 5 shows cumulatively the percentage of predictions for which the relative error is below a threshold. The subject-independent model outperformed both baselines by a significant margin. However, the predicted VILR has still an error larger than 25% for 12% of the samples in the test set. The subject-dependent fails for only 3% of the examples.

### Computing Time

The mean calculation time for each prediction was 142 ms (2.3 GHz Intel Core i5), of which the majority (140 ms) is spent on estimating the ground contact time. Meaning a prediction of the VILR can be made within one foot contact (160 - 350 ms).

## Discussion

The overall aim of this study was to predict the VILR during overground running by creating performant machine learning models. Advanced signal processing was used to identify time-discrete features of the 3D acceleration waveforms that discriminate between subtle changes in running biomechanics. Machine-learned models were subsequently built to estimate the VILR and the performance (predictive accuracy, diagnostic ability) of those models were compared to a traditional approach. Two other machine learning techniques not discussed in this study were attempted, but gave unsatisfactory results. First, a data-driven deep recurrent neural network would require much more data than available to learn the complex relations between the tibial acceleration signals and VILR. Second, dynamic time warping was used as a tool for gait-curve matching, incorrectly assuming that runners with similar acceleration profiles have a similar VILR. Moreover, the feature engineering approach is preferable, since the learned models are interpretable (to a certain extend) and have a much lower computational cost.

The findings point out that applying machine learning to multiple 3D tibial acceleration features results in a more accurate prediction of the VILR than the frequently used APTA, which is a single time-discrete variable of tibial acceleration. Additionally, this prediction can be made in real-time, because the data pre-processing (i.e., filtering and feature construction) and prediction requires less calculation time than the typical duration of a single foot contact (∼250 ms).

Overall, the XGB models systematically outperformed the other learners, suggesting that the XGB model can cope more effectively with the large number of features or that the relationship among the features and target are non-linear (Hepp et al., 2016).

From a machine learning setting perspective, building a subject-dependent model resulted in the most accurate predictions compared to the subject-independent models. The difference in predictive performance between the subject-independent model and subject-dependent model may partially be explained by the fact that all runners of the second cohort wore their own habitual running footwear, which might influence the measured impact loading. This assumption is further reinforced by the fact that the performance of the subject-independent model can be further improved by incorporating subject-describing features (body weight and shoe type). However, the phenotypical variability and choice of footwear can only partly explain the differences in accuracy between a subject-dependent and independent model. Although all runners ran in a similar environment, the ranked order of variable importance for predicting the VILR is unique for each runner in a subject-dependent learned model. Moreover, we observed a large asymmetry between the average VILR for most subject’s left and right legs, suggesting that the subject-dependent models could be further improved by building separate models for both legs. However, in our study not mirroring the data resulted in a worse predictive accuracy due to the limited amount of data available for each subject.

The better predictive performance for a subject-dependent model compared to a subject-independent model is in line with previous findings described by Wouda et al. (2018) and Ahamed et al. (2019). However, our subject-independent model is more practical toward real-world applications. It is applicable to any runner, regardless of whether prior data is available about the respective runner, which makes this approach generalizable over different subjects. Supporting our hypothesis, the subject-independent XGB model still outperformed the linear APTA model in terms of prediction accuracy and diagnostic ability.

By incorporating multiple running speeds we were able to create a machine learning algorithm that is capable of making accurate predictions across a broad range of running speeds, making it more usable in practice. As a consequence of this design choice, we observe relatively high R^2^ scores for these models in comparison with previous research that considered a single running speed (Laughton et al., 2003; Pohl et al., 2009; Greenhalgh et al., 2012; Zhang et al., 2016) due to the restricted range effect (Bland and Altman, 2011): the inclusion of multiple speeds increases the range of the maximal VILR and makes it easier to see the global trend. However, this applies to all models discussed here and therefore does not affect the inter-model differences. For comparison, the evaluation metrics for all models trained on exclusively the most frequent running speed of 3.2 m⋅s^–1^ are provided as Supplementary Table B.

The VILR was predicted accurately, using a broad range of variables derived from filtered 3D accelerations. In order to screen runners on their VILR at a common training speed of 3.2 m⋅s^–1^ (e.g., identifying runners with a high VILR, during a simple overground running test without the need of an expensive force plate) the classification of runners on impact intensity is preferably done by estimating VILR by means of a machine-learned model instead of relying on the APTA only. Because VILR is the maximum increase in acceleration of the lower extremity and of the rest of the body during stance (Clark et al., 2017), the predictive accuracy may be further improved by adding trunk acceleration to the accelerometer-derived input data.

This study has several limitations. Firstly, we trained the models only on habitual rearfoot strikers. Since machine learning can only be used to memorize patterns that are present in the training data, the trained models can only be applied to other rearfoot strikers and our findings do not necessarily generalize to other foot strike patterns. Secondly, all data was recorded in a laboratory environment. Previous research identified significant variations in APTA or contact time among different running surfaces (Tessutti et al., 2012; Boey et al., 2017). Hence, the findings should be transferred with caution to running on other surfaces.

## Conclusion

This study proposes an advanced method to predict VILR during overground running by using only tri-axial shin mounted accelerometers derived data and an XGB machine learning approach. These algorithms, which incorporate time-continuous variables, are able to predict the VILR more accurately than currently possible using a time-discrete variable (e.g., APTA). Since these algorithms do not require significant computational power, they could be implemented on wearables worn by the runner in order to screen, monitor or provide biofeedback on the predicted VILR whilst running overground.

## Data Availability Statement

The datasets generated for this study are available upon request to the corresponding authors.

## Ethics Statement

The studies involving human participants were reviewed and approved by the Ghent University Hospital Ethical Committee. The participants provided their written informed consent to participate in this study.

## Author Contributions

RD, PR, PB, DC, VS, and JD conceived, designed, and coordinated the study. RD, JG, and PB collected original data. PR and JD developed the machine learning algorithms. RD, PR, PB, and JG participated in data analysis. RD and PR developed the figures, initially drafted the manuscript, and the other authors provided useful suggestions in the preparation of the final manuscript. All authors reviewed the manuscript and gave approval for publication.

## Conflict of Interest

The authors declare that the research was conducted in the absence of any commercial or financial relationships that could be construed as a potential conflict of interest.
