# Supplementary material for: Tibial Acceleration-Based Prediction of Maximal Vertical Loading Rate During Overground Running: A Machine Learning Approach
Source: Front Bioeng Biotechnol. 2020 Feb 4;8:33. doi: 10.3389/fbioe.2020.00033 (PMC7010603; doi:10.3389/fbioe.2020.00033)
Supplement: Supplementary file 1 [file Data_Sheet_1.PDF]

Table A. Acceleration signal features which achieve a higher Pearson correlation (r) with the maximal vertical instantaneous loading rate (VILR) than the absolute peak tibial acceleration (APTA). Features are ranked by Pearson correlation. Additionally, the predictive performance of each feature is reported as the mean absolute error (MAE) $\pm$ sd and R^2 scores for the estimation of the VILR by linear regression models using a single variable in the subject-independent model learning setting.

	**Feature**	**Parameters**	**MAE $\pm$ sd**	$\mathbf{R^2}$	**r**
1	First fixes a corridor given by the quantiles q_l and q_h of the distribution of the vertical component of the acceleration signal. Then calculates the average, absolute value of consecutive changes in acceleration inside this corridor	$q_l = 0.2$, $q_h = 0.8$	17.47 ± 7.98	0.71	0.82
2		$q_l = 0.2$, $q_h = 1.0$	17.58 ± 7.27	0.71	0.82
3		$q_l = 0.0$, $q_h = 0.8$	17.69 ± 8.29	0.70	0.82
4		$q_l = 0.0$, $q_h = 1.0$	17.76 ± 7.58	0.71	0.82
5		$q_l = 0.4$, $q_h = 0.8$	18.32 ± 8.61	0.69	0.82
6		$q_l = 0.4$, $q_h = 1.0$	18.32 ± 7.44	0.70	0.81
7	Variance of the vertical component of the acceleration signal	-	18.64 ± 7.85	0.68	0.81
8	Minimal standard deviation on the estimated linear trend of the vertical component of the acceleration signal using chunks of length l	$l = 5$	17.76 ± 7.46	0.69	0.81
9	Standard deviation on the vertical component of the acceleration signal	-	19.57 ± 7.95	0.66	0.80
10	Mean standard deviation on the estimated linear trend of the vertical component of the acceleration signal using chunks of length l	$l = 5$	18.06 ± 7.28	0.67	0.80
11	First fixes a corridor given by the quantiles q_l and q_h of the distribution of the vertical component of the acceleration signal. Then calculates the variance of consecutive changes in acceleration inside this corridor	$q_l = 0.2$, $q_h = 0.8$	18.34 ± 7.49	0.66	0.80
12		$q_l = 0.0$, $q_h = 0.8$	18.38 ± 7.65	0.65	0.79
13		$q_l = 0.4$, $q_h = 0.8$	18.71 ± 7.81	0.65	0.79

14	Standard deviation on the estimated linear trend of the vertical component of the acceleration signal	-	18.42 ± 7.30	0.65	0.79
15	Mean standard deviation on the estimated linear trend of the vertical component of the acceleration signal using chunks of length l	$l = 10$	18.48 ± 7.36	0.65	0.79
16	First fixes a corridor given by the quantiles q_l and q_h of the distribution of the vertical component of the acceleration signal. Then calculates the average, absolute value of consecutive changes in acceleration inside this corridor	$q_l = 0.6$, $q_h = 0.8$	18.99 ± 8.27	0.65	0.79
17	The q quantile of the vertical component of the acceleration signal	$q = 0.8$	19.99 ± 7.50	0.64	0.78
18	Max standard deviation on the estimated linear trend of the vertical component of the acceleration signal using chunks of length l	$l = 5$	18.61 ± 7.14	0.65	0.78
19	First fixes a corridor given by the quantiles q_l and q_h of the distribution of the vertical component of the acceleration signal. Then calculates the variance of consecutive changes in acceleration inside this corridor	$q_l = 0.2$, $q_h = 1.0$	19.14 ± 7.16	0.64	0.78
20		$q_l = 0.0$, $q_h = 1.0$	19.17 ± 7.29	0.64	0.78
21		$q_l = 0.4$, $q_h = 1.0$	19.36 ± 7.31	0.64	0.78
22	The sum over the absolute value of consecutive changes in the vertical component of the acceleration signal	-	20.84 ± 8.64	0.62	0.77
23	The q quantile of the vertical component of the acceleration signal	$q = 0.9$	20.53 ± 7.74	0.62	0.77
24	First fixes a corridor given by the quantiles q_l and q_h of the distribution of the vertical component of the acceleration signal. Then calculates the variance of consecutive absolute changes in acceleration inside this corridor	$q_l = 0.0$, $q_h = 0.8$	19.45 ± 7.61	0.63	0.77
25	The third order autocovariance of the vertical component of the acceleration signal using lag l	$l = 1$	19.59 ± 7.39	0.62	0.77

26	First fixes a corridor given by the quantiles q_l and q_h of the distribution of the vertical component of the acceleration signal. Then calculates the variance of consecutive changes in acceleration inside this corridor	$q_l = 0.6$, $q_h = 0.8$	19.46 ± 7.87	0.62	0.77
27	The third order autocovariance of the vertical component of the acceleration signal using lag l	$l = 2$	19.64 ± 7.38	0.61	0.76
28	Complexity-Invariant Distance (CID) of the vertical component of the acceleration signal	-	20.29 ± 8.16	0.62	0.76
29	The absolute energy of the vertical component of the acceleration signal	-	20.89 ± 8.50	0.62	0.76
30	The third order autocovariance of the vertical component of the acceleration signal using lag l.	$l = 3$	19.75 ± 7.39	0.60	0.76
31	Variance on the intercept of the estimated linear trend of the vertical component of the acceleration signal using chunks of length l.	$l = 50$	19.38 ± 6.95	0.60	0.76
32	Absolute Peak Tibial Acceleration (APTA)	-	21.07 ± 8.13	0.60	0.75
