# Supplementary material for: Tibial Acceleration-Based Prediction of Maximal Vertical Loading Rate During Overground Running: A Machine Learning Approach
Source: Front Bioeng Biotechnol. 2020 Feb 4;8:33. doi: 10.3389/fbioe.2020.00033 (PMC7010603; doi:10.3389/fbioe.2020.00033)
Supplement: Supplementary file 2 [file Data_Sheet_2.PDF]

Table B. Mean absolute error (MAE) ± sd, coefficient of determination R^2 scores and effect sizes of MAE's versus the axial peak tibial acceleration (APTA) baseline for the estimation of the vertical instantaneous loading rate (VILR). These results only consider data from the trials performed at the most frequent running speed of 3.20 $m.s^1$. Linear Regression with Elastic Net regularization (EN), Linear Regression with Least Absolute Shrinkage and Selection Operator regularization (LASSO) and Gradient Boosted Regression Trees (XGB) in the subject-independent and subject-dependent learning settings.

Model	MAE $[BW.s^{-1}]$	R^2	d_{rm}	Effect size
**Subject-independent** (without subject-describing features)				
APTA	18.12 ± 9.02	0.4890	/	/
LASSO	13.62 ± 8.12	0.6813	0.3374	Medium
EN	13.81 ± 8.16	0.6749	0.3477	Medium
XGB	13.67 ± 7.63	0.6987	0.3751	Medium
**Subject-independent** (with subject-describing features)				
APTA	17.32 ± 9.25	0.4716	/	/
LASSO	13.21 ± 7.93	0.6601	0.3517	Medium
EN	13.69 ± 8.37	0.6365	0.3420	Medium
XGB	13.14 ± 7.23	0.6843	0.4011	Medium
**Subject-dependent**				
APTA	9.10 ± 4.26	0.8334	/	/
LASSO	11.68 ± 9.29	-0.6325	0.0487	Small
EN	10.41 ± 7.40	0.0017	0.0225	Small
XGB	7.78 ± 2.77	0.8842	0.1934	Small
